# “Our Tradition Our Enemy”: A Qualitative Study of Barriers to Women’s HIV Care in Jimma, Southwest Ethiopia

**DOI:** 10.3390/ijerph17030833

**Published:** 2020-01-29

**Authors:** Hailay Gesesew, Pamela Lyon, Paul Ward, Kifle Woldemichael, Lillian Mwanri

**Affiliations:** 1 Public Health, Flinders University, Adelaide 5042, Australia; hailushepi@gmail.com (H.G.); lillian.mwanri@flinders.edu.au (L.M.); 2 Epidemiology, College of Health Sciences, Mekelle University, Mekelle, Ethiopia; 3 Southgate Institute for Health, Society ad Equity, College of Medicine & Public Health, Flinders University, Adelaide 5042, Australia; pamela.lyon@internode.on.net; 4 Epidemiology, Institute of Health, Jimma University, Jimma, Ethiopia; kifle.wmichael@gmail.com

**Keywords:** barriers, delayed HIV diagnosis, discontinuation, Ethiopia, HIV care continuum, interventions, qualitative, tradition, women

## Abstract

Evidence exists that suggests that women are vulnerable to negative HIV treatment outcomes worldwide. This study explored barriers to treatment outcomes of women in Jimma, Southwest Ethiopia. We interviewed 11 HIV patients, 9 health workers, 10 community advocates and 5 HIV program managers from 10 institutions using an in-depth interview guide designed to probe barriers to HIV care at individual, community, healthcare provider, and government policy levels. To systematically analyze the data, we applied a thematic framework analysis using NVivo. In total, 35 participants were involved in the study and provided the following interrelated barriers: (i) Availability— most women living in rural areas who accessed HIV cared less often than men; (ii) free antiretroviral therapy (ART) is expensive—most women who have low income and who live in urban areas sold ART drugs illegally to cover ART associated costs; (iii) fear of being seen by others—negative consequences of HIV related stigma was higher in women than men; (iv) the role of tradition—the dominance of patriarchy was found to be the primary barrier to women’s HIV care and treatment outcomes. In conclusion, barriers related to culture or tradition constrain women’s access to HIV care. Therefore, policies and strategies should focus on these contextual constrains.

## 1. Introduction 

Human immunodeficiency virus (HIV), which can lead to significant impairment of the body’s resistance to infection and malignancy with development of autoimmune deficiency syndrome, led to one of the most devastating epidemics of the late 20th century [1]. Ethiopia is one of the countries hardest hit by the HIV/AIDS epidemic, from which women have suffered more significantly than men. While the male-to-female ratio of the Ethiopian population is almost equal (101:100), the prevalence of HIV among females (1.2%) is twice as high as in men (0.6%), and 33% higher than the overall prevalence in the entire population (0.9%) [2]. The prevalence rate of HIV among Ethiopian women with concurrent sexual partners (five or more) is even higher, being 6% [2]. In a 12-year retrospective cohort study to assess the complete HIV care continuum at a clinic dispensing antiretroviral therapy (ART) in Jimma, southwest Ethiopia, we found that 59% (3119 of 5299) of HIV patients on ART were females, implying a higher burden of HIV among this segment of the population [3]. Furthermore, we found that delayed presentation for HIV care [4], discontinuation from ART [5], immunologic failure [6], and HIV-related mortality [3] were higher among females than males. The performance of Ethiopian women relative to the UNAIDS 90-90-90 treatment targets is also low [7]. The UNAIDS 90-90-90 targeted at diagnosing 90% of people living with HIV, providing ART to 90% of those diagnosed with HIV, and achieving viral suppression for 90% of patients receiving treatment. Based on our study, the performance of women in Southwest Ethiopia was only 36-55-66 [8,9], which is substantially below the UNAIDS target.

Evidence shows that reasons for the high burden of HIV among women included personal factors (e.g., denial, mental illness, drug use, lack of trust, perceived health status, misconception) [10,11,12,13,14], social factors (e.g., stigma and discrimination, preference for traditional medicine) [10,15,16,17,18,19,20,21,22], financial factors (e.g., cost for medications for opportunistic infections, visiting health care facilities) [10,11,16,23], geographic and transportation factors (e.g., distance, transportation availability) [10,24,25,26,27,28], and barriers within the health system (e.g., length of appointments, waiting time) [10,29,30,31]. In addition, global literature revealed that socio-cultural issues such as gender roles, norms, and disempowerment appeared to be leading factors both in potential risk of HIV infection and against effective HIV care and treatment in women [32]. For example, Carael and colleagues stated, “women are noted to be more vulnerable and susceptible biologically, economically, and culturally than men” [33]. In general, culture is known to play a pivotal role in determining health at the individual, family, and community levels, but these effects tend to be exaggerated in Africa, where an individual’s behavior is profoundly affected by the values of extended family and community [34]. This has significant implications for sexual behavior and HIV/AIDS prevention and control efforts [34]. 

To reduce the prevalence of HIV and improve treatment outcomes among women in Ethiopia, several interventions or programs have been implemented to prevent mother-to-child HIV transmission Options including Options A, B, and B+ [35]; establish support organizations such as National Network of Positive Women Ethiopians [36]; and institute provider-initiated HIV testing and counseling, voluntary HIV counseling and testing [37], free service delivery at the point of care [38,39], case management [40], patient information systems [40], community mobilization and provision of care support services [40], the “3 by 5” initiative [41], Getting to Zero [42], Treat all [43], and the UNAIDS “90-90-90” targets [44]. 

Despite these programs, women remain at a higher risk of HIV infection and poor treatment outcomes. Yet limited evidence exists concerning why women in Ethiopia are so highly over-represented among those late to present for HIV care and most at risk to interruption or discontinuation of treatment. Moreover, such studies rarely explore the cultural context of intervention strategies and programs that may present potential barriers to participation, to say nothing of the multiple levels at which these may present. The qualitative study described in this paper was designed to explore as comprehensively as possible the barriers to HIV care and treatment for women in Jimma, Southwest Ethiopia.

## 2. Materials and Methods

The study was conducted using face-to-face, in-depth interviews among four different groups of study participants in Jimma Zone, Southwest Ethiopia. We interviewed 11 patients with HIV, 9 health workers, 10 community advocates and 5 top level program managers. Table 1 describes the sample size of each group and the institutions with which they are associated. 

We included patients who had history of ART discontinuation as well as those with a history of good treatment adherence throughout the care continuum. We purposefully recruited patients after they received service at the clinic. The HIV care provider distributed information about the study for patients to consider before deciding to participate or not. If they agreed, the participants were sent to a separate unlabeled room for interview. The health workers, community advocates and HIV program managers also were recruited purposefully. An invitation letter to target groups requesting assistance in recruiting study participants was posted on noticeboards in relevant units or departments, distributed in staff meetings, and circulated to mailing lists under the auspices of unit or department heads. The letter included the researcher’s mobile number. Volunteer participants were asked to report to a temporary office or send an SMS text for interview. The interviewer introduced himself to study participants through an introduction letter, which included an information sheet and a consent form to be signed before the interview started. The purposive criteria, to recruit the above study participants, was to consider gender, professional and stakeholders mix to get saturated information. 

We prepared an in-depth interview guide consisting the following constructs: a) Barriers to HIV care and treatment at individual level (knowledge, experience, expectations, attitudes, beliefs and disclosure); b) barriers at the community level (care and support, stigma and discrimination, and preferences for traditional healing); c) barriers within the healthcare system (quality of care, interaction with HIV health care providers, referral and linkage, logistics, availability, administration and capacity building); and d) barriers at the policy level (health policy, HIV/AIDS policy, health care financing, guidelines and standards). We made audio recordings of the semi-structured interviews, and made hand-written field notes immediately following each session. Each interview was fully transcribed verbatim and the transcripts were notated to clarify words, phrases and sounds:

(i) Words in parenthesis indicate possible hearings; for example, “(hospital)”;
(ii) Capitals excepting acronyms or those at the beginning of lines indicate loud sounds relative to the surrounding talk; for example, “NO NO”;
(iii) Numbers in parentheses show elapsed time in silence tenths of seconds; for example, “No (.2)”;
(iv) Punctuation indicates speaker’s intonation; for example, “Do you know what they do?”;
(v) Words under single quotation represent locals; for example, ‘Tsebel’.

We conducted both deductive and inductive analyses in order to establish a priori and emerging categories. We performed thematic framework analyses in tandem with data collection. Shortly after performing each interview, the researcher read the verbatim transcript and field note several times, and followed by performing transcriptions from the audiotaped interviews. Through this process we identified significant statements to generate initial codes for categories and themes and to further develop these as well as and sub-headings [45,46,47]. The detail coding process was published elsewhere [9]. Finally, we presented the themes and codes included under each team, and interpreted the illustrative quotes which supported the themes. Illustrative quotes are added while presenting the themes where “Pho” and “PHC” are quotes from patient participant from hospital and health center respectively, “HWHo” is a quote from health worker participant from a hospital, and “Admin.” is a quote from a participant from HIV program manager group. 

In order to establish validity of the data and display conflicting views, we triangulated narratives of findings from individuals, health workers, community representatives, and administrators. We used several techniques to ensure the trustworthiness, i.e., credibility, transferability, dependability, and reliability of data [48,49]. We used NVivo 12 Mac (QSR international Pty Ltd., Doncaster, Victoria, Australia, 2018) software to prepare transcripts, explore transcribed data, code empirical indicators, develop themes through building categories and nodes or code drops into the generated theme, and visualizing outcomes. 

Ethical approval was obtained from the Social and Behavioral Research Ethics Committee of Flinders University (Project No: 7698) and the Institutional Review Board of Jimma University (Ref. No: IHRPG/878/2017). Additional permissions and written consent were also acquired from the participating institutions and study participants prior to interview. We assured each study participant that the information gathered would be treated confidentially.

## 3. Results and Discussion

### 3.1. Characteristics of Study Participants

Thirty-five (35) participants, of whom 23 were females and 12 were males, and representing 10 institutions were interviewed between October 2017 and January 2018. The median age for HIV patients was 33 years, 40 years for HIV care providers, 32 years for community advocates and 35 years for HIV care administrators. The substantial majority of study participants (22, 63%) were followers of the Ethiopian Tewahedo Orthodox Church, a branch of Christianity, followed by Muslims (7, 20%) and Protestants (6, 17%). With the exception of one patient, all patients and community advocates had completed primary school, while all HIV care providers and HIV care administrators had completed college and above. Five of the 11 patients (45%) had histories of lost-to-follow up, i.e., missing one or more appointments after starting ART. The median period that patients had been on ART, which ranged from 2–16 years, was approximately nine years. Patients reported travelling a median time of 30 min (between 5 min to 2 h) to reach an ART clinic. The median work experience of community advocates was eight years (3–12 years), HIV care providers six years (2–12 years), and HIV care administrators in the HIV sector three years (2–12 years). 

### 3.2. Gender Differences on Barriers to HIV Care and Treatment

The study identified four themes in terms of barriers to receiving and continuing HIV care and treatment, which constrained women more than men. Barriers related to (i) availability, (ii) the paradoxical perception that free ART is expensive, (iii) the fear of being seen by others (stigma), and (iv) the role of tradition, which directly or indirectly affected the other three. 

#### 3.2.1. Availability

Lack of access to or availability of HIV care—for example, lack of services or personnel—was mentioned as one of the barriers to women’s HIV care. This was specifically mentioned by HIV patients and community representatives, although it was complemented by a considerable number of HIV care providers and HIV program managers. Most respondents stated that females suffer more than males in areas where services do not exist or are less available and accessible, particularly in rural areas. As one female HIV patient observed:

“A woman who doesn’t disclose her HIV status face difficulty to go to health center to collect drugs. Because she has no reason to tell her husband where she went, why she did not feed the children, why she didn’t do the housework, etc.…. All these are the responsibility of women in village. If the health facility is near, she could quickly go and collect the pills and back to her children and cattle. But the transport (and its cost) still matters.”

(PHC—03)

In addition to demonstrating female vulnerability to poor availability of HIV services due to the lack or cost of transport, the quote suggests not only that women are afraid to disclose their HIV status to their husband and family, but also that they fear even being seen visiting an HIV clinic by family members or people who know the family. Study participants made it clear that husbands expect their wives to look after the house and children, and the animals. Husbands in rural areas also closely monitor household expenditure, which means HIV-positive wives may not get enough money to cover transportation costs to the clinic even if transportation is available. 

These factors revealed by the interviews not only corroborated but also helped to explain the findings of our retrospective cohort study, published elsewhere, which showed that Ethiopian women are more likely to delay getting tested for HIV, commence ART late [4], and, if started, may interrupt or discontinue treatment [5]. Studies have already shown that rural residence, accompanied by inadequate infrastructure (including transportation), are associated with delayed HIV diagnosis and ART linkage, as well as ART attrition [25,26,27,28]. 

#### 3.2.2. The Paradoxical Perception that ART is Expensive

The codes included in theme are lack of food to initiate the treatment and unaffordable associated costs for other treatments (e.g., cotrimoxazole) and visiting health practitioners, selling ART drugs in black markets, fear of ART stock out and subsequently purchasing ART medicines from these illicit markets. Although ART is universally free in Ethiopia as a matter of government policy, the current study found the perceived high cost of ART was another barrier to HIV care, particularly among women. This was only mentioned by HIV patients and a few participants of the other groups. The roots of this belief, as identified in this qualitative study, branched in many directions, some of them unexpected. They touched on related beliefs about food intake required by ART treatments, the reality of female economic dependence on men, the need to sell ART drugs on the black market for additional household income, and even concern that civil unrest in Ethiopia will lead to drug shortages that will have to be met on these illicit markets. 

The belief that expensive food is needed while taking ART drugs comes from the participant’s idea that HIV medicine is strong, and if the body is not strengthened with nutritious food, such as milk, meat, and egg, the medicine will not work—or worse, the patient will be harmed without it. If people feel that they do not have adequate food, either they will not commence the treatment or if have already commenced it, they will probably stop. For example, a female patient with HIV said:

“Yes, people believe that when you start ART, your appetite will increase and you need to have adequate food; otherwise you will die if you take it on empty stomach. That’s why, some HIV patients prefer not to start ART if they feel they don’t have enough money to buy good food and you see this stuff more in women HIV patients.”

(PHo—08)

The verbatim statement shows that some people assume that ART is so physiologically depleting that it requires consumption of costly foods to maintain ordinary strength. Such foods are very expensive, however, and many Ethiopians cannot afford them. Consequently, HIV-positive people who are unable to afford these foods sometimes opt not to initiate treatment. Such views are particularly prevalent among women, our interviews showed. These findings support those of other studies that reported nutritional insufficiency as an obstacle to receiving ART or remaining in HIV care [23,28]. For example, a Nigerian study, where the majority of the participants were females, found that farmers with HIV who could not afford to purchase meat were less likely to continue treatment.

Another factor revealed in the interviews that contributed to the paradoxical perception that free ART drugs are expensive, was the women’s economic dependence on men. Several study participants noted that because women depend on their husbands or male family members for money, they often cannot afford the additional, non-ART-related costs of HIV infection that arise as a result of being medically treated for HIV. Our finding provides support for other studies suggesting that the inability to bear costs associated with HIV care acts as an obstacle to HIV-positive patients seeking or maintaining treatment [11,16,23]. 

One of the unexpected findings of this study, given that ART is free throughout Ethiopia, was the operation of an illicit market in ART drugs, which reportedly is supplied by patients, including some who register at and collect medications from more than one clinic, and from private clinics in the capital city of Addis Ababa. One patient reported:

“You are letting me speak what I don’t want to speak (smiling …). Let’s talk frankly. You know that there is a civil conflict in some places in Oromia, Gambela (provinces of the study setting) …… Many people with HIV have fear, and I myself have a great fear. There is a fear that the pharmacy may be robbed or the medications may be taken away to other places. So, if you have money and such fear, why wouldn’t you buy? I know I will get them in a black market either from patients or private clinics in Addis (Addis Ababa, Capital city of Ethiopia). By the way, people used to buy ART drugs for treating kidney failure.”

(PHC—02)

The connotation that HIV patients are registered in one ART clinic or more to sell ART drugs hiddenly could lead to at least double reporting on the number of HIV patients, and biases the HIV incidence and prevalence of the nation. Conversely, those HIV patients who take non-prescribed medicine have numerous individual and public health implications. For example, they may develop severe acute and chronic side effects related with ART, and face drug–drug interaction problems and drug resistance. Asked why patients buy ART drugs from black markets, the answers were due to the fear of stigma, in order to send drugs to HIV-positive family members or friends living abroad, and the fear of the ART stock running out due to civil arrest. All these lead to poor HIV care and treatment outcomes.

Another interesting finding that the study participants mentioned was that there were “known unknown” scenarios. A cross analyses among the four-target audiences—HIV patients, health workers, community advocates, and program managers— showed that HIV patients knew ART was sold in illicit markets, but the HIV program managers and most HIV care providers and community representatives did not know about this issue. A HIV program manager revealed,

“ART for sale? This is my first time, but I will take a note. I am very happy for the information though. What I have observed is we have to have a check and balance system. We know patients sometimes don’t give us their real name and address. This may open to the enrolment here and there you are saying. It also makes tracing of lost patients very complex. I wish there is a uniform software to avoid the double reporting.”

(Admin.—02)

We can note from the above quote that the program managers were surprised by the issue. Thus, the issue of ART for sale is a hidden yet serious problem for both sellers and buyers. The issue of ART for sale in illicit markets is not limited to Ethiopia. A study from South Florida found that HIV medicines were for sale in black marketplaces, and reasons for this comprised having limited access to HIV care, to replace lost or ruined medicines, and to have a backup stock [50]. Similarly, a study from South Africa also reported this [51]. 

#### 3.2.3. Fear of Being Seen by Others

HIV-related stigma and the fear of being seen by others was a cross-cutting constraint throughout the entire continuum of HIV care, according to our interview subjects. This was mentioned by all the four groups of study participants where the great majority of each group also raised the issue. Although reported as a barrier to HIV care by the majority of members in all groups represented in the present study, women reported being affected by stigma more often than men. For instance, a women HIV patient stated that:

“People don’t want to be recognized as HIV-positive in their workplaces or other social gatherings. I remember a ridiculous scenario. There was a HIV-positive woman in a group of people eating porridge. One of them (from the HIV-negative women) pierced the container hiddenly in order to flow down the virus along with the cheese. Seeing this, why should one go to get tested and know HIV status? Why would you disclose your HIV status?”

(PHo—09)

The quote illustrates the extent to which stigma to negatively influence HIV diagnosis and ART retention. It also demonstrates how stigma leads to misconception, for example, that HIV can be transmitted via eating together. Unlike with other illnesses, HIV patients also tend to be isolated by other people, who assume that the HIV-positive are somehow “cursed”, the study participants said. Fear of HIV-related stigma may be so strong that individuals who decide not to have their HIV status checked, much less begin treatment, nevertheless may visit private clinics for treatment of infections and other medical issues. This fear also causes some HIV patients to travel long distances to collect their medicine, despite the availability of local ART clinics, in order not to be recognized by their neighbors, thereby increasing the costs of free HIV treatment. For example, a health worker said:

“People, especially the women, who come from distant places such as Gambella (450 kms far from Jimma, the study setting) complain to take ART for four or five months in a single visit, as they don’t have money for transportation. They sometimes even use plane, if not safe by bus. If they don’t have that transportation cost, the option is to stop… so they are suffering.... but this doesn’t mean that there is no ART clinic in Gambella. Some others don’t want people to talk about them, about their taking ART regimens…NEVER.”

(HWHo—08)

The extreme fear of stigma even worries HIV patients about what will happen after their death. This was supported by the following quote from a patient. “I can tell you that nobody will care for my dead body except those people with HIV” said one patient (PHC—02). The profoundly negative effects of HIV stigma on the entire continuum of HIV care shown by our study—even today, nearly half a century since the HIV/AIDS epidemic began—confirms the findings of numerous studies [15,16,17], some of which have also shown that women are more greatly affected by this stigma than men [52]. 

#### 3.2.4. The Role of Tradition

What became clear during all of the interviews conducted in this qualitative study is that Ethiopian culture, which is deeply patriarchal, presents the greatest barrier to women seeking HIV care and continuing ART treatment, at least in the Jimma region of the country’s southwest. The three types of barriers described above, which relate to greater vulnerability to HIV stigma, luck of access to HIV care services and lack of access to independent monetary resources—are all fundamentally linked to the constraints placed by culture and religion on women’s roles in Ethiopian society. That role is, first and foremost, defined by family life. The low cultural status of women, including permissiveness toward domestic violence against women, is also reflected in common Ethiopian proverbs from all provinces in the nation. Three examples, from each of the major dialect regions of Ethiopia, are provided below.

1. Tigrigna (ትግርኛ): ሰበይትን ኒሁግን እናወቐጥካ። (phonetics): Sbeitin nihugin enaweqetak. Translation: A woman and a niger-seed shall be hit.
2. Amharic (አማርኛ): ሴት ሲበዛ ጎመን ጠነዛ። (phonetics): Set sibeza gomen teneza. Translation: When there are too many women, the cabbage will be ruined.
3. Afan Oromo (Afaan Oromo): Beekkumsi dubartii jilbaa (phonetics): Bekumsi dubarti jilba gadi. Translation: The knowledge of the women is below the knee.

In rural societies in particular, men are assumed to be the breadwinner and women are assumed to work in the kitchen, thus women’s access to education is also limited [53]. In our study many interview subjects observed that traditional healing practices, such as drinking water from holy places or that has been otherwise blessed, were widely considered a real alternative to seeking medical treatment, including for HIV. Reasons given included that traditional healers could be accessed easily, commanded more respect than their modern medical counterparts, were better trusted by patients, and were less expensive. Consistent to other studies [21,22], our study revealed that women visited traditional healers more than men, which increased their vulnerability to HIV complications even more. As also found in other studies [54,55,56], patriarchal culture and social pressure, which limit female autonomy, make things worse. One female health worker observed:

“… Females are the ones who frequently go to traditional healings like Tsebel (drinking holy water) and then after, stop drugs. In addition, a husband in our tradition is very dominant in the society; so, if he orders his wife not to go to health centres, she will stop. How ridiculous tradition we have? In fact, husbands are happy to send their wives to holy places.”

(HWHo—08)

Interpreting the above quote, women have little autonomy, meaning that they are much more vulnerable to censure based on their behavior. The fact that women visit traditional practitioners more than men implies a difficulty to access HIV care services either due to lack of available services or their husbands influence. 

Traditional healing practices, which in Ethiopia are closely related to an indigenous orthodox Christianity, may also act as a barrier to effective HIV treatment through misconceptions arising from religious belief. Some religious leaders do not allow foreign material (e.g., ART) to be brought into holy places, such as Tsebel and churches, as these foreign objects are believed to curse the holy place, according to some of the interview subjects. In addition, some said that some priests believe ART is the demon’s work, and its use is believed to test or interrogate God. Pastors in one Protestant church, who believe that praying to God will eliminate HIV, were reported to have told their followers to stop taking ART drugs, an admonition which carries potentially direct negative consequences for the health of HIV+ parishioners. A female HIV+ patient who sought a cure via holy water said,

“… while you drink the Holy Water, the virus will be removed in the form of diarrhea once your sprit communicated with God. The Muslims come here too to share this blessing. The Protestants go to their pastor. Everybody wants to try and communicate with his creator.”

(PHC—03)

Interpreting the verbatim statements, simultaneous use of traditional and modern medicine was also compromised by spiritual leaders. Poor collaboration between the two health care practices was also demonstrated. Studies from Ethiopia [16] and other African countries [18,19,20] confirm the role of traditional healing practices as an important barrier to women’s HIV care. 

The study had some limitations: (i) The fact that female interviewees were interviewed by a male interviewer may have affected the data collection process, although the interviewer knew the local language, norm and concerns raised by women; (ii) the sample size for the HIV care program managers (n=5) and the number of female HIV patients (n = 9) were small; nevertheless, the overall sample size (n = 35) was within the acceptable range for qualitative studies, and 66% of all participants were females. Furthermore, most findings from the four target groups complemented each other; (iii) the study participants were only recruited from Jimma, leaving it questionable as to whether more contextual factors were explored. Nevertheless, the data saturation was reached. 

## 4. Conclusions

The great majority of study participants in the present study revealed that the patriarchal culture of the society was found to be the main cause of causes and drivers of poor HIV care and treatment outcomes for women. In addition, HIV-related stigma was also found to be a cross-cutting barrier to the entire HIV care continuum. Lack of access to HIV care and treatment services, and preferences for visiting traditional healers were also obstacles to good HIV care and treatment outcomes that affected more women than men. ART for sale in illicit markets, another barrier to HIV care that affected more women than men, was known by patients with HIV and some HIV care providers, but not by every top-level program manager. As these are contextual constraints, our recommendations are for policy and strategic considerations that target addressing these factors as part of HIV management in Ethiopia. We recommend further big national studies with large sample sizes to investigate these barriers at a national level. It is also important to further research other contextual factors related to structural inequalities facing women.

## Figures and Tables

**Table 1:** Sample size and study participants, Jimma, Southwest Ethiopia, 2017/8.

Name of Group	Number of Participants		Source of Participants
	Male	Female	
Patients with HIV	2	9	Jimma University Teaching Hospital and Jimma Health center
HIV care providers	4	5	Jimma University Teaching Hospital and Jimma Health center
Community advocates ^a^	3	7	Jimma town, and selected kebeles ^b^ from Jimma Zone
HIV program managers	3	2	Jimma Zone Health Department, Jimma Town Health Office, Jimma HIV/AIDS prevention and control office, Organization Service for Social Aid, and the International Centre for AIDS Care and Treatment Program in the Southwest regions

^a^ Refers to HIV patients’ association, religious groups, ‘Idir’ (local association at the community level), women associations from selected kebeles, and community health extension workers; ^b^ Kebele is the lowest administrative unit in Ethiopia.

## References

[1] Moore R.D. (2011). Epidemiology of HIV Infection in the United States: Implications for Linkage to Care. Clin. Infect. Dis..

[2] CSA, ICF (2018). Ethiopian Demographic Health Survey 2016.

[3] Gesesew H., Ward P., Hajito K., Mwanri L. (2018). Early mortality among children and adults in antiretroviral therapy programs in Southwest Ethiopia, 2003–2015. PLoS ONE.

[4] Gesesew H.A., Ward P., Woldemichael K., Mwanri L. (2018). Late presentation for HIV care in Southwest Ethiopia in 2003–2015: Prevalence, trend, outcomes and risk factors. BMC Infect. Dis..

[5] Gesesew H.A., Ward P., Woldemichael K., Mwanri L. (2017). Prevalence, trend and risk factors for antiretroviral therapy discontinuation among HIV-infected adults in Ethiopia in 2003–2015. PLoS ONE.

[6] Gesesew H.A., Ward P., Woldemichael K., Mwanri L. (2018). Immunological failure in HIV-infected adults from 2003 to 2015 in Southwest Ethiopia: A retrospective cohort study. BMJ Open.

[7] UNAIDS (2017). UNAIDS Data 2017.

[8] Gesesew H., Ward P., Hajito K., Mwanri L. (2017). P3.90 HIV care continuum outcomes in Ethiopia: Surrogates for UNAIDS 90–90–90 targets for ending HIV/AIDS. 2017 STI & HIV World Congress.

[9] Gesesew H.A. (2019). HIV Care Continuum in Jimma, Southwest Ethiopia: A Multiphase Mixed Methods Study. Ph.D. Thesis.

[10] Moneyham L., McLeod J., Boehme A., Wright L., Mugavero M., Seal P., Norton W.E., Kempf M.C. (2010). Perceived Barriers to HIV Care Among HIV-Infected Women in the Deep South. JANAC J. Assoc. Nurses Aids Care.

[11] Smillie K., Van Borek N., van der Kop M.L., Lukhwaro A., Li N., Karanja S., Patel A.R., Ojakaa D., Lester R.T. (2014). Mobile health for early retention in HIV care: A qualitative study in Kenya (WelTel Retain). AJAR.

[12] Li H., Wei C., Tucker J., Kang D., Liao M., Holroyd E., Zheng J., Qi Q., Ma W. (2017). Barriers and facilitators of linkage to HIV care among HIV-infected young Chinese men who have sex with men: A qualitative study. BMC Health Serv. Res..

[13] Lederman M.M., Cannon P.M., Currier J.S., June C.H., Kiem H.P., Kuritzkes D.R., Lewin S.R., Margolis D.M., McCune J.M., Mellors J.W. (2016). A Cure for HIV Infection: “Not in My Lifetime” or “Just Around the Corner”?. Pathog. Immun..

[14] Ngarina M., Popenoe R., Kilewo C., Biberfeld G., Ekstrom A.M. (2013). Reasons for poor adherence to antiretroviral therapy postnatally in HIV-1 infected women treated for their own health: Experiences from the Mitra Plus study in Tanzania. BMC Public Health.

[15] Marya Viorst G., Linda M.C., Charles M.C., Noelle R.L., Leo W., Monica G.R., Scott B., David C.P., Alexandra K., Amanda S.R. (2017). Using the multiphase optimization strategy (MOST) to optimize an HIV care continuum intervention for vulnerable populations: A study protocol. BMC Public Health.

[16] Miho S., Belkis W., Gabrielle O.M. (2016). Barriers to and Factors Facilitating Adherence to Antiretroviral Therapy from the Perspectives of Patients in Maqala City, Tigray Region, Ethiopia. Nilo-Ethiop. Stud..

[17] Porter K.E., Brennan-Ing M., Burr J.A., Dugan E., Karpiak S.E. (2017). Stigma and Psychological Well-being Among Older Adults With HIV: The Impact of Spirituality and Integrative Health Approaches. Gerontologist.

[18] Tso L.S., Best J., Beanland R., Doherty M., Lackey M., Ma Q., Hall B.J., Yang B., Tucker J.D. (2016). Facilitators and barriers in HIV linkage to care interventions: A qualitative evidence review. AIDS.

[19] Kagee A., Remien R.H., Berkman A., Hoffman S., Campos L., Swartz L. (2011). Structural barriers to ART adherence in Southern Africa: Challenges and potential ways forward. Glob. Public Health.

[20] Loeliger K.B., Niccolai L.M., Mtungwa L.N., Moll A., Shenoi S.V. (2016). Antiretroviral therapy initiation and adherence in rural South Africa: Community health workers’ perspectives on barriers and facilitators. Aids Care.

[21] Shewamene Z., Dune T., Smith C.A. (2017). The use of traditional medicine in maternity care among African women in Africa and the diaspora: A systematic review. BMC Complement. Altern Med..

[22] Gedif T., Hahn H.J. (2002). Epidemiology of herbal drugs use in Addis Ababa, Ethiopia. Pharmacoepidemiol. Drug Saf..

[23] Bezabhe W.M., Chalmers L., Bereznicki L.R., Peterson G.M., Bimirew M.A., Kassie D.M. (2014). Barriers and Facilitators of Adherence to Antiretroviral Drug Therapy and Retention in Care among Adult HIV-Positive Patients: A Qualitative Study from Ethiopia. PLoS ONE.

[24] Anyinam C. (1987). Availability, accessibility, acceptability, and adaptability: Four attributes of African ethno-medicine. Soc. Sci. Med..

[25] Pellowski J.A. (2013). Barriers to care for rural people living with HIV: A review of domestic research and health care models. JANAC.

[26] Hontelez J.A., Tanser F.C., Naidu K.K., Pillay D., Barnighausen T. (2016). The Effect of Antiretroviral Treatment on Health Care Utilization in Rural South Africa: A Population-Based Cohort Study. PLoS ONE.

[27] Joseph D.T., Lai Sze T., Brian H., Qingyan M., Rachel B., John B., Haochu L., Mellanye L., Marley G., Rich Z.C. (2017). Enhancing Public Health HIV Interventions: A Qualitative Meta-Synthesis and Systematic Review of Studies to Improve Linkage to Care, Adherence, and Retention. EBioMedicine.

[28] Agbonyitor M. (2009). Home-based care for people living with HIV/AIDS in Plateau State, Nigeria: Findings from qualitative study. Glob. Public Health.

[29] Mugavero M.J., Norton W.E., Saag M.S. (2011). Health Care System and Policy Factors Influencing Engagement in HIV Medical Care: Piecing Together the Fragments of a Fractured Health Care Delivery System. Clin. Infect. Dis..

[30] Roura M., Busza J., Wringe A., Mbata D., Urassa M., Zaba B. (2009). Barriers to sustaining antiretroviral treatment in Kisesa, Tanzania: A follow-up study to understand attrition from the antiretroviral program. Aids Patient Care Stds.

[31] Colvin C.J., Konopka S., Chalker J.C., Jonas E., Albertini J., Amzel A., Fogg K. (2014). A systematic review of health system barriers and enablers for Antiretroviral Therapy (ART) for HIV-infected pregnant and postpartum women. PLoS ONE.

[32] Moreno C.L. (2007). The relationship between culture, gender, structural factors, abuse, trauma, and HIV/AIDS for Latinas. Qual. Health Res..

[33] Carael M., Pitt D., Staugard F. (1996). Women, AIDS, and STDs in sub Saharan Africa: The impact of marriage change. AIDS and the Grassroots: Problems, Challenges, and Opportunities.

[34] Airhihenbuwa C.O., Webster J.D. (2004). Culture and African contexts of HIV/AIDS prevention, care and support. SAHARA J..

[35] WHO (2010). WHO. WHO Guidelines Approved by the Guidelines Review Committee. Antiretroviral Drugs for Treating Pregnant Women and Preventing HIV Infection in Infants: Recommendations for a Public Health Approach: 2010 Version.

[36] NNPWE National Network of Positive Women Ethiopians (NNPWE). https://www.nnpwe.org/.

[37] EHNRI, MoH (2012). HIV Related Estimates and Projections for Ethiopia.

[38] Assefa Y., Damme W.V., Mariam D.H., Kloos H. (2010). Toward Universal Access to HIV Counseling and Testing and Antiretroviral Treatment in Ethiopia: Looking Beyond HIV Testing and ART Initiation. Aids Patient Care Stds.

[39] Gilks C.F., Crowley S., Ekpini R., Gove S., Perriens J., Souteyrand Y., Sutherland D., Vitoria M., Guerma T., De Cock K. (2006). The WHO public-health approach to antiretroviral treatment against HIV in resource-limited settings. Lancet.

[40] Assefa Y., Alebachew A., Lera M., Lynen L., Wouters E., Van Damme W. (2014). Scaling up antiretroviral treatment and improving patient retention in care: Lessons from Ethiopia, 2005–2013. Glob. Health.

[41] WHO UNAIDS Treating 3 Million by 2005. Making it Happen. http://www.who.int/3by5/publications/documents/isbn9241591129/en/.

[42] UNAIDS Getting to Zero Selected as World AIDS Day Theme. http://www.unaids.org/en/resources/presscentre/featurestories/2011/november/20111101wadtheme.

[43] WHO (2015). Consolidated Guidelines on the Use of Antiretroviral Drugs for Treating and Preventing HIV Infection: What’s New?.

[44] UNAIDS (2014). UNAIDS 90-90-90: An Ambitious Treatment Target to Help end the AIDS Epidemic.

[45] Gale N.K., Heath G., Cameron E., Rashid S., Redwood S. (2013). Using the framework method for the analysis of qualitative data in multi-disciplinary health research. Bmc Med. Res. Methodol..

[46] Srivastava A., Thomson S.B. (2009). Framework Analysis: A Qualitative Methodology for Applied Policy Research. JOAAG.

[47] Ritchie J., Spencer L. (1994). Qualitative Data Analysis for Applied Policy Research.

[48] Lincoln Y., Guba E. (1985). Naturalistic Inquiry.

[49] Merriam S. (2009). Qualitative Research: A Guide to Design and Implementation.

[50] Tsuyuki K., Surratt H.L., Levi-Minzi M.A., O’Grady C.L., Kurtz S.P. (2015). The Demand for Antiretroviral Drugs in the Illicit Marketplace: Implications for HIV Disease Management Among Vulnerable Populations. Aids Behav..

[51] Larkan F., Van Wyk B., Saris A.J. (2010). Of Remedies and Poisons: Recreational Use of Antiretroviral Drugs in the Social Imagination of South African Carers. Afr. Sociol. Rev..

[52] Piscitelli P., Iolascon G., Argentiero A., Chitano G., Neglia C., Marcucci G., Pulimeno M., Benvenuto M., Mundi S., Marzo V. (2012). Incidence and costs of hip fractures vs strokes and acute myocardial infarction in Italy: Comparative analysis based on national hospitalization records. Clin. Interv. Aging.

[53] Emily G., Benjamin R., Tasmia B. (2014). An Investigation into the Barriers to Female Education in Link Ethiopia Schools.

[54] Dejene N.D. (2009). Gender and culture in southern Ethiopia: An ethnographic analysis of Guji-Oromo women’s customary rights. Afr. Study Monogr..

[55] Shroufi A., Mafara E., Saint-Sauveur J.F., Taziwa F., Vinoles M.C. (2013). Mother to Mother (M2M) peer support for women in Prevention of Mother to Child Transmission (PMTCT) programmes: A qualitative study. PLoS ONE.

[56] MacPherson P. (2013). Improving Linkage into HIV Care Among Adults in Blantyre, Malawi. Ph.D. Thesis.

